# Platelet Function, Role in Thrombosis, Inflammation, and Consequences in Chronic Myeloproliferative Disorders

**DOI:** 10.3390/cells10113034

**Published:** 2021-11-05

**Authors:** Lisa Repsold, Anna Margaretha Joubert

**Affiliations:** Department of Physiology, School of Medicine, Faculty of Health Sciences, University of Pretoria, Pretoria 0007, South Africa; annie.joubert@up.ac.za

**Keywords:** platelets, inflammation, thrombosis, myeloproliferative disorders

## Abstract

Platelets are conventionally defined as playing a vital role in homeostasis and thrombosis. This role has over the years transformed as knowledge regarding platelets has expanded to include inflammation, cancer progression, and metastasis. Upon platelet activation and subsequent aggregation, platelets release a host of various factors, including numerous pro-inflammatory factors. These pro-inflammatory factors are recruiters and activators of leukocytes, aiding in platelets’ immune regulating function and inflammatory function. These various platelet functions are interrelated; activation of the inflammatory function results in thrombosis and, moreover, in various disease conditions, can result in worsened or chronic pathogenesis, including cancer. The role and contribution of platelets in a multitude of pathophysiological events during hemostasis, thrombosis, inflammation, cancer progression, and metastasis is an important focus for ongoing research. Platelet activation as discussed here is present in all platelet functionalities and can result in a multitude of factors and signaling pathways being activated. The cross-talk between inflammation, cancer, and platelets is therefore an ideal target for research and treatment strategies through antiplatelet therapy. Despite the knowledge implicating platelets in these mentioned processes, there is, nevertheless, limited literature available on the involvement and impact of platelets in many diseases, including myeloproliferative neoplasms. The extensive role platelets play in the processes discussed here is irrefutable, yet we do not fully understand the complete interrelation and extent of these processes.

## 1. Platelet Morphology and Function

Platelets are known to lack a nucleus and are referred to as anucleate cells. Platelets are derived from the fragmentation of the cytoplasm from bone marrow megakaryocytes and, if not activated, circulate in the blood for approximately 10 days [1,2,3]. In addition to the bone marrow, studies have reported on the production of platelets from megakaryocytes in the lungs. These findings have shown that conventional knowledge of haematopoiesis is inadequate. Haematopoietic progenitors, including mature and immature megakaryocytes, are found in the lungs, and, in cases of thrombocytopenia, these progenitors can reconstitute blood platelet counts by repopulating the bone marrow. In fact, the biogenesis of platelets from megakaryocyte precursors in the lungs is so significant that up to 50% of the total platelet production originates from the lungs [4]. Platelets are conventionally defined as playing a vital role in hemostasis and thrombosis [1,2,3]. This role has, over the years, transformed as our knowledge regarding platelets has expanded to include their role in inflammation, cancer progression, and metastasis.

**Figure 1:**
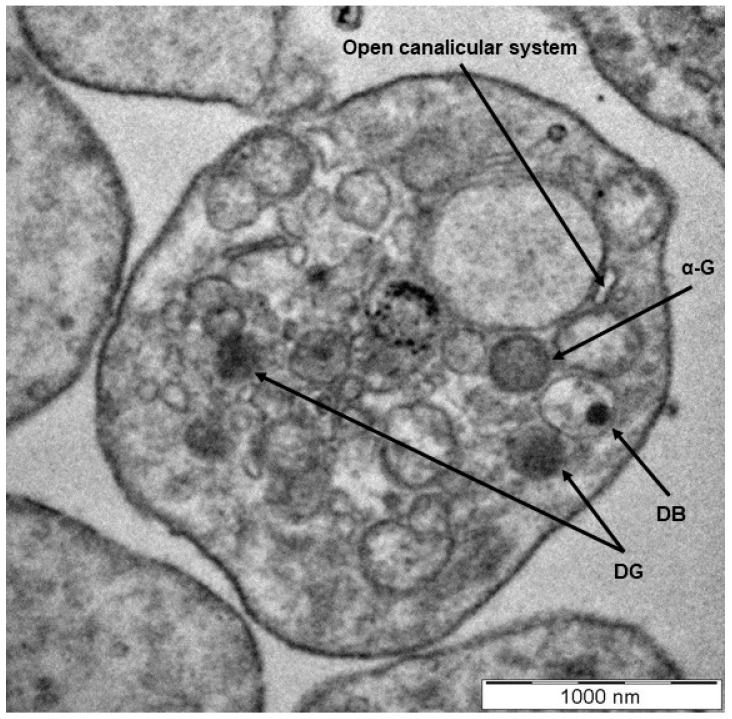
A transmission electron microscopy image showing the morphology of a healthy platelet. The discoid, compacted structure indicates a platelet at rest with clear visualization of the dense granules (DG) and dense bodies (DB), α-granules (α-G), and an open canalicular system (scale indicates 1000 nm) [7,8,9,10,11,12].

**Figure 2:**
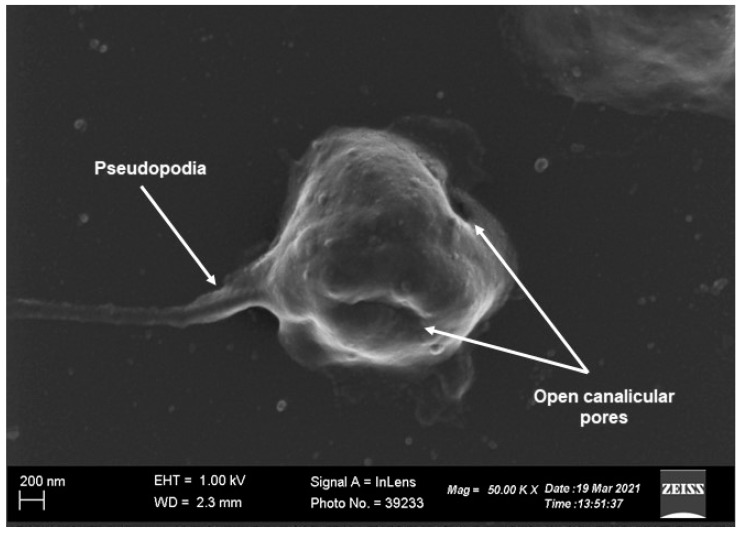
Scanning electron microscopy image of a platelet indicating normal morphology. The discoid, compacted structure of the platelet points to the platelet being at rest with well-defined open canalicular pores that communicate with the extracellular environment (scale indicates 200 nm) [7,8,9,10,11,12].

Platelets are the smallest circulatory cells, measuring 2 to 5 µm in diameter, and are biconvex, discoid discs that, when inactivated, contain open canalicular systems, an intricate array of membranes that communicate with the extracellular space, α-granules, dense granules, lysosomes, and mitochondria (Figure 1 and Figure 2) [1,2,3,5,6]. Upon platelet activation and subsequent aggregation, platelets release a host of various factors, including, but not limited to, fibrinogen, which is converted to fibrin to form blood clots, aiding in platelets’ characteristic function in thrombosis and wound healing.

## 2. Platelet Granule Constituents and Membrane Receptors

Platelets’ involvement in coagulation and the resulting formation of a platelet plug during damage to blood vessels is a result of a wide variety of factors released from the α-granules (Table 1), dense granules (Table 2), and lysosomes (Table 3) contained within the platelet. These factors are released upon activation signals relayed from membrane receptors (an overview of these receptors is presented in Table 4), which include agonists such as thrombin, adenosine diphosphate (ADP), thromboxane A_2_ (TXA_2_), and the phosphorylation of various target proteins, resulting in high-affinity ligand binding and platelet aggregation [13].

Advances in technology and research into platelet constituents and membrane receptors have enabled mapping of the platelet proteome through transcriptomic and proteomic determination. In these studies, for example, 1282 platelet proteins, 788 of which had not been reported on previously, have been identified [12,14]. Proteomics do, however, present with limitations, as the bioinformatics process and parameters of studies need to be identical for any comparisons to be drawn. In addition, several other limitations in proteomic experimentation and workflow exist, including biological variations in samples, sample storage, appropriate controls, sample amount, statistics, etc. Therefore, even though proteomic platelet determination is of significance, aiding in our knowledge of platelets’ biology and function, the limitations necessitate an integration of proteomic, transcriptomic, metabolomic, and bioinformatic fields in combination with clinical data to fully elucidate platelet function and to assist in identifying targets for antiplatelet therapy [12,14]. Table 1, Table 2, Table 3, Table 4 therefore provide an overview of a few principal platelet constituents and membrane receptors and ligands.

The most important of these receptors are the glycoproteins (GP), including the GPIb-IX-V complex aiding in platelet adhesion and the GPII-IIIa receptor facilitating platelet aggregation. When damage or injury occurs to the endothelial layer of blood vessels, components of the subendothelial connective tissue are exposed, including collagen [15]. The von Willebrand Factor (vWF) is subsequently released from α-granules of platelets and endothelial cells adjacent to the injury, allowing platelets to establish a connection to exposed collagen [14,25,26]. This connection is mediated through vWF by interaction to GPIb-IX-V complexes present on the platelet membrane, thus allowing adhesion via the GPIa-IIa receptor [14,26,27,28].

**Figure 3:**
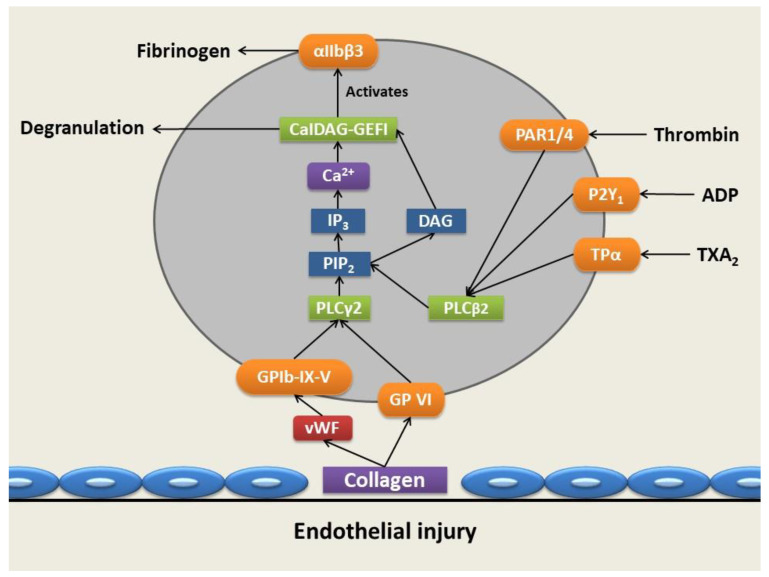
Platelet adhesion, activation, and aggregation following vascular injury. Upon vessel injury, collagen is exposed, allowing the binding of collagen to GPVI and the GPIb-IX-V complexes through von Willebrand Factor (vWF), allowing platelet adhesion via the GPIa-IIa receptor. This results in the activation of phospholipase Cγ2 (PLCγ2), which hydrolyzes phosphatidylinositol 4,5-bisphosphate (PIP_2_), producing 1,2-diacylglycerol (DAG) and inositol trisphosphate (IP_3_), the latter of which results in a cytoplasmic increase in calcium (Ca^2+^) ions. The increase in Ca^2+^ ion levels and DAG levels activates diacylglycerol-regulated guanine nucleotide exchange factor I (CaIDAG-GEFI) and, subsequently, the αIIbβ3 integrin, which exposes the fibrinogen binding site. The activation of protease-activated receptor 1 (PAR-1) and protease-activated receptor 4 (PAR-4) receptors is mediated through thrombin, while purinergic receptor (P2Y_1_) is activated by the binding of adenosine diphosphate (ADP) and thromboxane A_2_ (TXA_2_) receptors (TPRs)—TPα is activated by TXA_2_. Resulting activation of these receptors causes coupling via Gq to phospholipase C-beta2 (PLCβ2), also generating DAG and a further increase in Ca^2+^ cytosolic levels through phosphorylation of PIP_2_, facilitating platelet activation and degranulation (produced with Microsoft PowerPoint) [2,28,29,30,31,32,36,37,38].

To further strengthen the platelets’ adhesion to the site of vascular injury, GPVI also binds to collagen [29]. The binding of vWF to GPIb-IX-V and collagen to GPVI gives rise to an outside-to-inside transduction of signals and consequent platelet activation [29]. This results in the activation of phospholipase Cγ2 (PLCγ2) via a signaling cascade known as the signalosome. Following the activation of PLCγ2, it hydrolyzes phosphatidylinositol 4,5 bisphosphate to inositol trisphosphate (IP_3_) and 1,2-diacylglycerol (DAG), which is membrane-bound [29,30,31,32]. IP_3_ functions as a secondary messenger, which results in an efflux and corresponding cytoplasmic increase in Ca^2+^ from the dense tubular system (DTS) upon binding of IP_3_ to its receptor (IP_3_ receptor) on the DTS (Figure 3) [29].

The increase in Ca^2+^ ion concentration activates the diacylglycerol-regulated guanine nucleotide exchange factor I (CaIDAG-GEFI) that leads to the activation of the small G protein Ras-related protein 1 (Rap1). An activation complex is subsequently formed comprised of activated Rap1, talin, and kindlin3 that binds to the cytoplasmic tails of GPIIb/IIIa, resulting in the disruption of the clinch between the α- and β subunits, causing a conformational change that stimulates stretching of the extracellular domains, thus exposing the fibrinogen binding site [14,25,26,27,28,29,30,31,32,35]. Upon exposure of fibrinogen binding sites, fibrinogen binds hereto and results in the linkage of activated platelets through the formation of fibrinogen bridges, facilitating platelet aggregation [27].

In addition, thrombin activates its protease-activated receptor 1 (PAR-1) and protease-activated receptor 4 (PAR-4) by cleavage of the N-terminal of the consensus site [29]. Purinergic receptor (P2Y_1_), an ADP receptor, is also activated by the binding of ADP released from degranulating dense granules [25]. Activation of both of these receptors results in coupling to phospholipase C-beta2 (PLCβ2) via Gq as a main effector molecule that also generates DAG and IP_3_ and further increases Ca^2+^ cytosolic levels within the platelet-facilitating platelet activation and degranulation [28,29,30,31,32]. P-selectin plays a role in cohesion between platelets only after activation and also arbitrates communication with leukocytes to adhere to the platelet plug [15]. This takes place through the binding of P-selectin on the membrane of activated platelets with its receptor, P-selectin glycoprotein ligand 1 (PSGL1) found on leukocytes and endothelial cells, enabling platelet-leukocyte and platelet-endothelial cell interactions [29].

## 3. Platelets’ Role in Inflammation and Cancer

Our evolved knowledge of platelet function has, in recent years, focused on platelets’ roles in inflammation and cancer progression through angiogenesis and metastasis [39,40]. Platelets have been recognized as an extension of the cellular immune system and play a role in leukocyte functioning and the subsequent release of inflammatory signals [40,41,42,43]. In addition, platelets express Toll-like receptors (TLRs), which allow platelets to directly interact with microbial pathogens akin to the functioning of leukocytes [40,41,42,43]. Upon platelets’ detection of microbes through TLRs, platelets are activated and degranulated, releasing numerous pro-inflammatory factors. These pro-inflammatory factors are recruiters and activators of leukocytes, aiding in platelets’ immune regulating functions, which include the release of cluster of differentiation 154 (CD154) or CD40 ligand; CXC chemokine ligand (CXCL)-1, CXCL4, CXCL5, CXCL7, CXCL12, interleukin (IL)-8; and transforming growth factor (TGF)-β. The release of CD154 from platelets is the primary source of this molecule as part of the adaptive immune response and results in lymphocyte activation [39,40,41,42,43,44].

Neutrophil extracellular traps (NETs) function as part of the immune system by trapping and capturing circulating microbes, thereby limiting microbes’ transport and colonization throughout the body. Studies have shown that platelets adhering to neutrophils can result in NET formation (NETosis), and this serves as a platform for platelet docking and activation, which can result in thrombosis [40,41,42,43]. The expression of Toll-like receptor 4 (TLR4) on platelets is reported to result in NETosis in activated neutrophils, releasing histones 3 and 4 concurrently, which further activates platelets in a constant loop, promoting additional NET formation [45]. It is therefore apparent that the various platelet functions are interrelated; activation of the inflammatory function results in thrombosis and, moreover, in various disease states. This can result in worsened or chronic pathogenesis of certain diseases, including rheumatoid arthritis and cardiovascular diseases such as, atherosclerosis [40,41,42,43].

Another example of platelets’ involvement in disease progression that implicates the inflammatory and thrombotic functions of platelets discussed is cancer. The first report of platelets’ involvement in cancer was in 1865 by Armand Trousseau, who reported that distant platelet-induced venous thrombosis was caused by localized cancers [45]. Later on, in 1968, Gasic, Gasic, and Stewart reported that platelet-deficient mice with thrombocytopenia were protected against cancer metastasis [46]. Since then, research on the involvement of platelets in cancer has been well researched and reported on, including the incidence of tumor-induced platelet aggregation in pancreatic, colorectal, and kidney cancers. Platelets’ contribution to cancer can be summarized by the hallmarks of cancer they influence, which include (1) sustaining proliferative signals, (2) resisting cell death, (3) supporting cancer stem cells, (4) inducing angiogenesis, and (5) metastasis and evading immune detection [40,41,42,43].

Tumor-induced platelet aggregation is associated with an increase in cancer metastasis and is primarily caused by the release of thrombin, TXA_2_, and ADP. The release of thrombin, TXA_2_, and ADP from tumor cells results in the activation of platelets [45]. In particular, ADP is known to activate platelets through P2Y_1_ and P2Y_12_ receptors, facilitating further ADP release from platelets’ dense granules, which activate nearby platelets, resulting in a cascade of tumor-induced platelet activation [45].

TGFβ is a known immunosuppressive cytokine released from platelet α-granules, resulting in tumors and tumor cells evading recognition by the immune system and subsequent apoptosis [44]. In addition, the release of pro-inflammatory factors recruits leukocytes to primary and metastatic tumor sites and promotes metastasis through the formation of NETs [44]. In addition to TGFβ, further growth factors are released from platelet α-granules, including the vascular endothelial growth factor (VEGF) and the platelet-derived growth factor (PDGF), inducing tumor growth, angiogenesis, and tumor neovascularization. These growth factors have been reported to be increased in the plasma of patients with haematological malignancies and solid tumors [40,41,42,43].

Platelets’ implication in cancer-associated inflammation, thrombosis, angiogenesis, and metastasis has become a focus for research through targeting of platelets for the possible treatment of cancer [47]. Platelet-affecting drugs, such as aspirin, have been reported to have anticancer or cancer-preventative effects and anti-metastatic properties and are known to inhibit platelet function [40,41,42,43,47]. The active ingredient of aspirin, acetylsalicylic acid, is known to inhibit cyclooxygenase (COX)-enzymes-1 and -2 (COX-1 and COX-2), which are responsible for the formation of TXA_2_, resulting in the inhibition of platelet activation and aggregation and therefore has been postulated to have an anticancer, protective effect [44,47]. This mechanism of action is shared with non-steroidal anti-inflammatory drugs (NSAIDs). COX-1 is expressed by platelets, while COX-2 is expressed by endothelial and tumor cells, including breast, bladder, lung, gastric and pancreatic cancers. There is therefore uncertainty whether the anticancer effects of aspirin are as a result of platelet inhibition or due to the inhibition of COX in tumor cells [40,41,42,43]. However, it may be a combined effect of both these processes that presents a significant target for platelet-affecting drugs, such as aspirin, as a combined anticancer treatment with conventional cancer treatments such as chemotherapy, radiation therapy, or immunotherapy [40,41,42,43].

Multiple clinical trials in patients with adenomas, adenomatous polyposis, non-polyposis colorectal cancer, gastrointestinal cancer, and pancreatic cancer showed a decrease in the incidence and risk of these cancers when patients were treated with low-dose aspirin daily. In addition, daily low dose aspirin also revealed a decrease in the risk of metastasis in breast, lung, and prostate cancers [48]. The anti-metastatic effect of aspirin through targeting of platelets has been mentioned to be due to the inhibition of platelet COX-1 activity, and this platelet-dependent inhibition can be summarized to be a result of the targeting of various phases of metastasis which include tumor-induced platelet aggregation, endothelial cell activation, tumor cell adhesion to the endothelium, recruitment of macrophages, and formation of the pre-metastatic niche [48].

Additional antiplatelet drugs are of interest and include clopidogrel and prasugrel (a P2Y_12_ target), which irreversibly inhibit ADP receptors; tricagrelor (a P2Y_12_ target), which reversibly inhibits ADP receptors; abciximab, eptifibatide, and tirofiban (integrin αIIbβ3 target), which inhibit aggregation through blocking integrin αIIbβ3; and vorapaxar (a PAR1 target), which inhibits coagulation through blocking thrombin receptors [48,49]. Novel targets for antiplatelet therapies include targeting GPVI and GPIb-V-IX; PI3Kβ inhibitors; and tyrosine kinase inhibitors, which, in platelets, affect spleen tyrosine kinase (SYK), bruton tyrosine kinase (BTK), sarcoma family kinases (SRC), and MAPK signaling [48,49]. These drugs are of particular interest in the field of cardiovascular diseases to prevent thrombus formation and lack proper long-term research and follow-up in an oncology/haematology setting as part of translational medicine and treatment for patients. These drugs as targets for cancer treatment are, however, limited by the number of antiplatelet therapies available and concerns regarding bleeding risks in these patients [2,36,48,49].

## 4. Platelets’ Role in Myeloproliferative Disorders

Haematological malignancies are a class of neoplasms or cancers that affect the myeloid or lymphoid cell lines. Haematological malignancies and their neoplastic phenotypes are derived from changes or mutations in the differentiation process of haematopoiesis, resulting in distinct changes in the factors governing survival and proliferation [50]. Mutations in myeloid cell lines result in acute and chronic myelogenous leukemia, myelodysplastic syndromes, and myeloproliferative disorders, whereas mutations in the lymphoid cell lines result in lymphomas, lymphocytic leukemia, and myeloma [50].

Literature and research in the pathogenesis and disease progression of haematological malignancies have mostly focused on myeloid and lymphoid populations. The process of normal haematopoiesis results in platelet formation from the differentiation of common myeloid progenitors and, subsequently, megakaryocytes. In haematological malignancies, normal haematopoiesis is altered through the transformation of haematopoietic stem cells [51,52]. These transformations, depending on the specific type of neoplasm, are known to occur in both lymphoid and myeloid progenitor cells and therefore have been postulated to also result in abnormalities of platelet differentiation from the same transformed myeloid progenitor cells and megakaryocytic lineages [51,52]. Despite this, and, in addition to frequent reports of platelet count abnormalities, including thrombocytosis, thrombocythemia, and thrombohaemorrhagic complications, the involvement and impact on platelets in haematological malignancies are not fully understood [53,54].

Thrombosis and related complications associated with platelet activation have been reported as one of the leading causes of morbidity and mortality in chronic myeloproliferative neoplasms [40,43,55]. Chronic myeloproliferative neoplasms are therefore of particular interest and consist of chronic myeloid leukemia (CML)and Philadelphia-negative myeloproliferative neoplasms, which are defined to be clonal haematopoeitic stem cell disorders identified through increased production of myeloid progenitors and mature blood cells [55]. In this class of haematological malignancies, three distinct disorders can be categorized (1) essential thrombocythemia (ET), identified by megakaryocyte proliferation and thrombocytosis; (2) polycythemia vera (PV), recognized by increased erythrocytes and erythroid expansion; and (3) primary myelofibrosis (PMF), distinguished by bone marrow fibrosis and increased dysplastic megakaryocytes and granulocyte progenitors [55,56,57,58,59]. These myeloproliferative neoplasms are characterized by hyperactivation of janus kinase 2 (JAK2)-signaling as a result of mutations in three specific genes: *JAK2*, *calreticulin* (*CALR*), and *myeloproliferative leukemia virus* (*MPL*). The most frequent molecular mutation present in myeloproliferative neoplasms is the *JAK2V617F* mutation present in more than 95% of PV patients and 50–60% of ET and PMF patients, followed by *CALR* mutations and *MPL* mutations, respectively [55,56,57,58,59]. In addition, a less common mutation in *JAK-2 exon 12* has been attributed as the causative mutation in PV.

**Figure 4:**
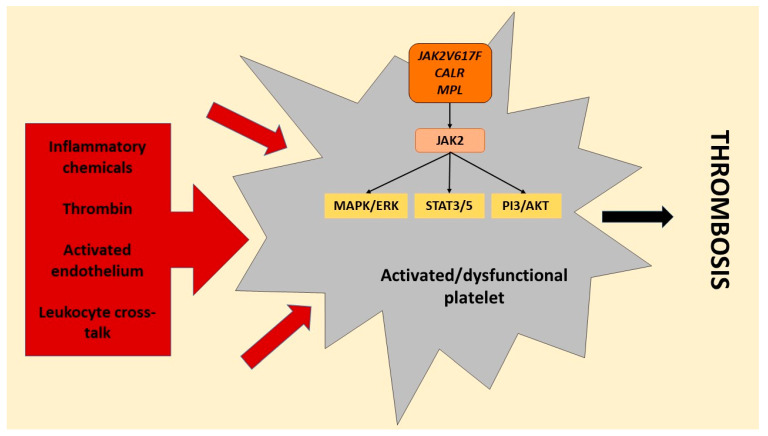
Platelet activation signals in myeloproliferative neoplasms. Platelets in myeloproliferative neoplasms are affected by the causative mutations (janus kinase 2 (JAK2)/*JAK2V617F*, *calreticulin* (*CALR*), and *myeloproliferative leukemia virus* (*MPL*)) in the haematopoietic stem cells, resulting in dysfunctional platelets prone to activation with hyperactive JAK2 signaling. The mutations in *JAK2*, *CALR* and *MPL* hyperactivate downstream signaling of JAK2, which activates mitogen-activated protein kinases/extracellular signal-regulated kinases (MAPK/ERK) and phosphoinositide 3-kinase/serine/threonine kinase Akt (PI3/AKT) and signals the transducer and activator of transcription 3/5 (STAT3/5). In addition, platelets are also activated by various external triggers affecting both clonally mutated and unmutated platelets, including the release of a multitude of inflammatory chemicals (cytokines and chemokines), thrombin, interaction with activated endothelium, and cross-talk between platelets and activated leukocytes, resulting in hyperactivated platelets. These intrinsic and extrinsic causal signals culminate in a thrombotic state in myeloproliferative neoplasms (produced with Microsoft PowerPoint) [55,56,57,58,59].

The process that results in activated platelet-related thrombosis in myeloproliferative neoplasms includes intrinsic platelet abnormalities due to transformed haematopoietic stem cell function that brings about overactive JAK2-dependent signaling and various extrinsic factors. These include cellular interaction with activated leukocytes, endothelial cells, and soluble mediators, including TXA_2_, which further prompt platelet activation in platelet populations derived from clonal cells in addition to megakaryocytes that are not derived from the malignant clone (Figure 4) [30,33,45]. The transformed haematopoietic stem cells and resulting megakaryocytes with altered gene expression have been reported to bring about circulating platelets with changed haemostatic and inflammatory functions. Impaired haemostatic function has been described to include decreased P-selectin, CD36, and fibrinogen binding [55,56,57,58,59].

Furthermore, platelets of patients with chronic myeloproliferative neoplasms demonstrate an increased platelet turnover, resulting in a greater quantity of newly formed platelets with heightened platelet reactivity, contributing to the platelets’ hyper-activated state and subsequent thrombosis [55,56,57,58,59]. The resulting platelet activation subsequently leads to increased GPIIbIIIa activation and platelet aggregate formation. Additionally, various inflammatory mediators, including pro-inflammatory chemokines, such as RANTES (CCL5), platelet factor 4 (PF4) (CXCL4), and IL-8, are released from the activated platelets, which further enhances the pro-inflammatory and pro-thrombotic loop in chronic myeloproliferative neoplasms [55,56,57,58,59].

Reports of *JAK2*-positive patients have indicated an increase in platelet activation markers, leukocytes, and endothelial activation, as well as circulating microparticles [55,56,57,58,59]. The crosstalk between the various platelet functionalities becomes clear in this context, implicating platelet activation, resulting in platelet-mediated inflammation and thrombosis, which explains the high incidence of thrombosis-related morbidity and mortality in myeloproliferative neoplasms [55,56,57,58,59]. Antiplatelet therapies discussed earlier therefore pose a risk in myeloproliferative neoplasms, as patients with specific subgroup mutations, including *CALR*-positive-ET patients and patients with extremely high platelet counts, are at higher risk of bleeding when receiving low-dose aspirin [55,56,57,58,59]. In this population of neoplasms, alternative antiplatelet strategies are an option, including statins, which have been shown to produce anti-inflammatory effects and curb platelet, endothelial, and leukocyte activation. The effectiveness of statins as a potential treatment in this setting, however, needs to be established clinically in myeloproliferative neoplasms [55,56,57,58,59].

Abnormalities in the granule constituents of platelets of patients with leukemia and myeloproliferative disorders, including CML, have been reported, specifically storage pool deficiency, which is the decreased formation of dense granules [55,60]. Storage pool deficiency resulting in abnormalities of adenosine triphosphate (ATP), ADP, calcium, serotonin, and pyrophosphate expression is a result of anomalies in platelet-dense granules formation. These anomalies have been attributed to chromosomal changes in the transformed myeloid progenitor cells and megakaryocytic lineages during haematopoiesis [60].

It is apparent that platelets’ role in the human body is widespread, extending far beyond hemostasis and thrombosis to include inflammation, cancer progression, and metastasis [40,61]. Its role and contribution in a multitude of pathophysiological events during these processes is an important focus for ongoing research, especially since platelet function is thoroughly interrelated. Platelet activation, as discussed here, is present in all platelet functionalities and can result in a multitude of factors and signaling pathways being activated, including thrombosis, inflammation, and metastasis [40,61]. Targeting platelet activation through existing anti-platelet therapies or through research into innovative new treatments and a full understanding of the mechanisms of platelet-mediated inflammation and platelet-mediated cancer progression should be an avenue to explore in future research [40,61]. The extensive role platelets play in the processes discussed here is irrefutable, yet we do not fully understand the complete cross-talk, interrelation, and extent of these processes.

## 5. Conclusions

Platelets provide physical and mechanical support to cancer cells to elude the immune system and metastasize, thereby supporting the tumorigenic process [1,2,3]. In addition, platelets play an integral role in inflammation and thrombosis that exacerbates the pathogenesis of various diseases, including cancer. Platelet activation is integral to these processes and mediates various pro-cancerous effects through the release of various soluble mediators from platelet granules. The cross-talk between inflammation, cancer, and platelets is therefore an ideal target for research and treatment strategies through antiplatelet therapy. In addition, studies to elucidate how platelets regulate between their pro- and anti-tumor, inflammatory, and thrombotic signaling is of particular interest to aid in novel treatment strategies. Despite the knowledge implicating platelets in these mentioned processes, there is, nevertheless, limited literature available on the involvement and impact of platelets in many diseases, including myeloproliferative neoplasms. This cross-talk or overlap between platelets’ functions in various diseases pathogenesis stands to be understood to aid clinicians in developing individualized treatment options. Due to the fact that platelets play an important role in inflammation, cancer, and tumor development, as discussed here, their role and potential influence in cancer progression is of clinical significance and warrants further investigation into possible novel therapeutic strategies targeting platelet function to improve patient outcomes.

## Figures and Tables

**Table 1:** Overview of factors released from platelet α-granules [15,16,17,18,19,20]. The α-granule degranulates upon activation of the platelet, which may occur in cases of stress, disease, or normal haemostatic function in formation of the platelet plug.

	Factors Released from α-Granules
Adhesion proteins	Fibrinogen Fibronectin Thrombospondin Vitronectin von Willebrand factor
Chemokines	Connective tissue activating peptide III CXC chemokine ligand (CXCL)-1, 2, 3, 4, 5, 6, 7, 8, 12 and 16 Macrophage inflammatory protein 1α Neutrophil-activating peptide 2 Platelet factor 4 Regulated upon activation, normal T cell expressed and presumably secreted (RANTES) β-Thromboglobulin
Coagulation factors	Factor V Factor VII Factor XI Factor XIII Kininogens Plasminogen Protein S
Growth factors	Angiopoietin-1 and 2 Basic fibroblast growth factor Endothelial cell growth factor Epidermal growth factor Hepatocyte growth factor Insulin-like growth factor 1 Interleukin-2, 3, 4, 6, 7, and 8 Interleukin-β Platelet-derived growth factor Transforming growth factor β Vascular endothelial growth factor A & C
Immunoglobulins	Immunoglobulin A (IgA) Immunoglobulin E (IgE) Immunoglobulin G (IgG) Immunoglobulin M (IgM)
Protease inhibitors	C1-inhibitor Plasminogen activator inhibitor-1 Platelet inhibitor of factor XIα Platelet-derived collagenase inhibitor Protease nexin-II/amyloid β-protein precursor Tissue factor pathway inhibitor α-1-proteinase inhibitor α_2_-antiplasmin α_2_-antitrypsin α_2_-macroglobulin
Proteoglycans	Histidine-rich glycoprotein Neutrophil-activating peptide 2 Platelet basic protein Serglycin
Other	Albumin Glycoprotein Iα/Multimerin

**Table 2:** Overview of factors released from platelet dense granules [15,16,17,18,19,20,21]. These factors within the dense granules are also released upon activation of the platelet specifically following binding of ADP and collagen to the platelet.

	Factors Released from Dense Granules
Amines	Adrenaline Dopamine Histamine Noradrenaline Serotonin
Bivalent cations	Calcium (Ca^2+^) Magnesium Polyphosphates Pyrophosphate
Nucleotides	Adenosine 5′-diphosphate Adenosine 5′-triphosphate Guanosine 5′-diphosphate Guanosine 5′-triphosphate

**Table 3:** Overview of factors present in lysosomes [15,16,17,18,19,20]. Lysosomes are known to also degranulate upon platelet activation and contain various digestive enzymes that are activated under acidic conditions.

	Factors Present in Lysosomes
Acid Proteases	Acid phosphatase Arylsulphatase Carboxypeptidases A & B Cathepsins D & E Collagenase Proline carboxypeptidase
Glycohydrolases	Heparinase α-D-glucosidase α-D-mannosidase α-L-arabinosidase α-L-fucosidase β-D-fucosidase β-D-galactosidase β-D-glucoronidase β-D-glucosidase β-glycerophosphatase β-N-acetyl-D-hexosaminidase

**Table 4:** Overview of platelet granule components (receptors and/or ligands) [14,22,23,24,25,26,27,28,29,30,31,32,33,34,35,36,37,38]. The receptors found on platelet membranes are of significance in the platelets’ primary function, which is to hinder haemorrhage after vascular injury. These receptors aid in the platelets’ adhesion, activation, and, finally, aggregation.

	Receptors Present on Platelet Membrane
C-type lectin receptor family (selectins)	Cluster of differentiation 72 (CD72) Cluster of differentiation 93 (CD93) C-type lectin 2 (CLEC-2) P-selectin (CD62P)
Immunoglobulin receptors	Cluster of differentiation 23 (CD23/FcεRI) Cluster of differentiation 32 (CD32/FcγRIIA) Cluster of differentiation 47 (CD47) Endothelial cell-selective adhesion molecule (ESAM) G6B Glycoprotein VI (GPVI) Intercellular adhesion molecule 2 (ICAM-2) Junction adhesion molecule 1 (JAM-1) Junction adhesion molecule 3 (JAM-3) Platelet and T cell activation antigen 1 (PTA-1) Platelet endothelial cell adhesion molecule (PECAM-1) TREM-like transcript 1 (TLT-1)
Integrins (glycoproteins)	α2β1 (GPIa-IIa—collagen-binding receptor) α5β1 (fibronectin receptor) α6β1 (laminin binding cell adhesion receptor) αIIbβ3 (GPIIb/IIIa (CD41/CD61)) αLβ2 (leukocyte function-associated antigen 1) α*v*β3 (vitronectin receptor)
Leucine-rich repeats receptors	GPIb-IX-V complex Toll-like receptors (TLR) 1, 2, 4 and 6 Matrix metalloproteinases (MMP) 1, 2, 3, 9 and 14
Lipid receptors	Lysophosphatidic acid receptor (LPL-R) Platelet-activating factor (PAF) receptor Sphingosine-1-phosphate receptors
Other membrane receptors	C3-specific binding protein (CD46) Cluster of differentiation 100 (CD100) Cluster of differentiation 40 ligand (CD40L) Collectin receptor (C1q) Extracellular matrix metalloproteinase inducer (EMMPRIN) (CD147) Galectin receptors Glutamate receptors GPIIIb (CD36) Liver X receptors (LXR) Lysosomal-associated membrane protein 1 (LAMP-1) Lysosomal-associated membrane protein 2 (LAMP-2) P2X purinoceptor 1 (P2 × _1_) (ion channel receptor) Peroxisome proliferator-activated receptor γ (PPARγ) P-selectin glycoprotein ligand 1 (PSGL-1) Semaphorin 3A Tight junction receptors Tumor necrosis factor (TNF)
Tetraspanins	Cluster of differentiation 53 (CD53) Cluster of differentiation 9 (CD9) Lysosomal membrane-associated glycoprotein-3 (LAMP-3) (CD63)
G protein-coupled receptors	A2_a_-adenosine C-C chemokine receptor 1 (CCR1) C-C chemokine receptor 3 (CCR3) C-C chemokine receptor 4 (CCR4) C-X-C chemokine receptor 1 (CXCR1) C-X-C chemokine receptor 4 (CXCR4) Dopamine receptors P2Y_1_ and P2Y_12_ (guanosine triphosphate-coupled protein receptor) Platelet factor 4 Prostaglandin D_2_ receptor (DP_2_), Prostaglandin E_1_ receptor (EP), Prostaglandin E_2_ receptor (EP), Prostaglandin F_2_ receptor (FP), Prostaglandin I_2_ receptor (IP) Protease-activated receptor (PAR) 1, 2, 3 and 4 Serotonin (5-HT_2A_) TXA_2_ receptor (TP) V_1a_ vasopressin β2-adrenergic Β-Thromoglobulin
Tyrosine kinase receptors	c-MPL (thrombopoietin receptor) (CD110) Insulin receptors Platelet-derived growth factor receptors Tyrosine kinase with immunoglobulin and epidermal growth factor homology-1 receptors (Tie-1)

## Data Availability

Data sharing is not applicable to this article, as no datasets were generated or analyzed during the current study.
